# Analysis of symptoms and their potential associations with e-liquids’ components: a social media study

**DOI:** 10.1186/s12889-016-3326-0

**Published:** 2016-07-30

**Authors:** Qiudan Li, Yongcheng Zhan, Lei Wang, Scott J. Leischow, Daniel Dajun Zeng

**Affiliations:** 1 The State Key Laboratory of Management and Control for Complex Systems, Institute of Automation, Chinese Academy of Sciences, Beijing, 100190 China; 2 Department of Management Information Systems, University of Arizona, Tucson, AZ 85721 USA; 3 Mayo Clinic, Scottsdale, AZ 85259 USA

**Keywords:** e-cigarette, e-juice, e-liquid, Flavors, VG, PG, Nicotine, Symptoms, Reddit, Social media

## Abstract

**Background:**

The electronic cigarette (e-cigarette) market has grown rapidly in recent years. However, causes of e-cigarette related symptoms among users and their impact on health remain uncertain. This research aims to mine the potential relationships between symptoms and e-liquid components, such as propylene glycol (PG), vegetable glycerine (VG), flavor extracts, and nicotine, using user-generated data collected from Reddit.

**Methods:**

A total of 3605 e-liquid related posts from January 1st, 2011 to June 30th, 2015 were collected from Reddit. Then the patterns of VG/PG distribution among different flavors were analyzed. Next, the relationship between throat hit, which was a typical symptom of e-cigarette use, and e-liquid components was studied. Finally, other symptoms were examined based on e-liquid components and user sentiment.

**Results:**

We discovered 3 main sets of findings: 1) We identified three groups of flavors in terms of VG/PG ratios. Fruits, cream, and nuts flavors were similar. Sweet, menthol, and seasonings flavors were classified into one group. Tobacco and beverages flavors were the third group. 2) Throat hit was analyzed and we found that menthol and tobacco flavors, as well as high ratios of PG and nicotine level, could produce more throat hit. 3) A total of 9 systems of 25 symptoms were identified and analyzed. Components including VG/PG ratio, flavor, and nicotine could be possible reasons for these symptoms.

**Conclusions:**

E-liquid components shown to be associated with e-cigarette use symptomology were VG/PG ratios, flavors, and nicotine levels. Future analysis could be conducted based on the structure of e-liquid components categories built in this study. Information revealed in this study could be utilized by e-cigarette users to understand the relationship between e-liquid type and symptoms experienced, by vendors to choose appropriate recipes of e-liquid, and by policy makers to develop new regulations.

## Background

The electronic cigarette (e-cigarette) market has grown rapidly in recent years. Sales of e-cigarettes in the United States, currently estimated to be a $1.5 billion market, are expected to grow 24.2 % per year through 2018 [1]. Discussions of e-cigarette benefits, risks, and effects on health have become a hot topic [2, 3]. E-liquid, or e-juice, contains the substance inhaled by vapers, that is, e-cigarette users, and is considered as the main component in e-cigarettes. It is a mixture of propylene glycol (PG), vegetable glycerine (VG), flavor extracts, and nicotine [4]. PG is an organic glycerol made from propylene oxide, a petroleum byproduct, while VG is a natural organic glycerol made from vegetable oil, which comes from palm and/or coconut oil [5]. On one hand, PG and VG are used as the carrier to deliver the nicotine and flavor when vaporized into steam, on the other hand, they can provide the clouds of vapor. Various blends ranging from 100 % VG to 100 % PG are available. VG produces more vapor than PG, and offers a slight sweetness. PG provides more “throat hit” and usually carries flavor more strongly [4, 6–8]. The flavors are usually natural or artificial flavor concentrates generally advertised as safe. They tend to fall into a few categories: Fruits, Beverages, Sweet, Nuts, Cream, Menthol, Seasonings, and Tobacco. Tobacco companies have successfully marketed traditional tobacco products to youth by using flavor varieties. For instance, a study found that menthol and candy-like flavors increased little cigars’ and cigarillos’ appeal to starters by masking the heavy cigar taste [9], which might be a potential factor to result in youth use. Although the U.S. Food and Drug Administration (FDA) has banned fruit, candy, and clove flavored cigarettes since 2009, for the concerns of special appeal for children [10], flavors are still widely used for e-cigarettes. Bahl et al. found e-cigarette refill fluids were cytotoxic due to chemicals used to flavor fluids, but not nicotine; Cinnamon Ceylon was one of the products that contained the chemicals [11]. Behar et al. got enlightened by the previous research and tested eight cinnamon-flavored e-liquids, most of which were cytotoxic. Cinnamaldehyde and 2-methoxycinnamaldehyde were identified highly cytotoxic from the fluids [12]. Tierney et al. revealed 13 out of 30 e-cigarette fluids had more than 1 % by weight flavor chemicals, which was sufficiently high for inhalation exposure by vaping to be of toxicological concern [13]. In another study, menthol flavor e-liquids were found to relieve the craving for tobacco in recent quitters, possibly by obtaining a better throat hit, which is the feeling of smoke hitting back of the throat [14]. These studies indicated that e-cigarette flavors could be dangerous but attractive, which should be carefully studied by researchers.

All the elements in e-liquids form the unique e-cigarette vaping experience, which produces several health-related effects and responses. However, causes of symptoms and the impacts from e-liquids on health remain uncertain. In this paper, we will examine e-liquid components and symptoms based on user-generated data from social media.

Social media such as Facebook, Twitter, and YouTube have recently become a significant platform for health surveillance and social intelligence [15, 16]. E-cigarettes can be studied based on the user-generated data collected from such social media platforms [2, 3].

As one of the most popular forums in the world, Reddit has great influence and a huge number of user groups. As of June 28, 2015, Reddit had 163,966,958 unique visitors hailing from over 212 different countries, viewing a total of 7,086,828,967 pages according to the statistical data published by Reddit [17]. Since 2008, users have been allowed to create a community (called “subreddit”) where they can discuss interesting topics with each other. Some public health research has been done based on Reddit datasets. Pavalanathan and Choudhury used Reddit to study mental health [18], while Arthur used Reddit to track the outbreak of Ebola in 2014 [19]. There are many publicly available posts about components of e-liquids, user experiences with recipes for e-liquid mixtures, and feelings and symptoms caused by different e-liquids, which have the potential to influence e-cigarette and flavor related attitudes, choices, and behaviors [20].

Despite the growing number of literature of e-cigarette on YouTube, Twitter and Facebook, there are no published studies to date that have systematically mined components of e-liquid, user experience, and symptoms in the use of the e-cigarette utilizing data from Reddit. Given the potential of Reddit to promote e-cigarette use through user-generated content or by advertising, this study aims to gain a systematic understanding of the relationships among the components of e-liquid, user experiences of different ingredient (PG, VG, Nicotine, flavor) combinations, and symptoms with their potential reasons and solutions by analyzing e-cigarette e-liquid-related posts from Reddit. The data-driven findings mined from social media could possibly benefit regulatory agencies like FDA so that they can develop a better understanding of e-cigarette use.

## Methods

### Data collection

We collected e-cigarette related posts on Reddit from January 1st, 2011, to June 30th, 2015. In previous research, a wide range of data was collected based on several keywords related to e-cigarette. Some additional rules were applied to make sure that data was accurate and relevant [2, 3, 20]. Similar to a previous approach [20] we used 7 keywords in our search:“electronic cigarettes”, “e-cigarettes”, “ecigarettes”, “e-cigs”, “flavor”, “flavors”, and “e-juice” Several subreddits were returned and we chose the top 10 in ranking provided by Reddit: /r/electronic_cigarette, /r/ecigclassifieds, /r/ejuice, /r/Vaping101, /r/ejuice_reviews, /r/EJuicePorn, /r/DIY_eJuice, /r/ecig_vendors, /r/Vaping, /r/E_cigarette. Two methods were used to retrieve posts from these subreddits: 1) keywords search, 2) ranking by relevance, hot spot, importance, up-to-date information, and reply count. Hot spot was given by Reddit search engine, and calculated by comprehensively considering of the number of browsing, comments, upvotes, and downvotes of a specific post. Using these strategies, our dataset contained 493,994 posts on Reddit. Secondly, we obtained a total of 27,638 unique e-cigarette flavor-related posts using 29 flavor keywords, which could be retrieved from a previous research [20]. Finally, to analyze the relationship between symptoms and e-liquids’ components, VG and PG were further used as keywords to obtain a total of 3605 unique e-cigarette, e-liquid related posts, which were published by 2394 unique users.

### Data analysis

To gain a systematic understanding of e-juice components and their relationships, the following data analysis processes were carried out. Posts were classified into eight flavor categories based on our previous study: fruits, cream, sweet, tobacco, menthol, beverages, seasonings, and nuts [20]. With the help of the flavor classification framework, we could group the posts based on flavor categories.

We then used keywords VG and PG as filters to identify the distribution of VG/PG ratio in different categories of flavors. Typically, users in Reddit discussed all kinds of VG/PG ratios. The common ratios are shown in Table 1. We manually classified them into 5 categories: high VG, balanced but high VG, balanced, balanced but high PG, and high PG. The patterns of VG/PG distribution among different flavors were analyzed.

**Table 1:** Common VG/PG ratios and categories

Category	Number of posts	VG/PG ratio
High VG	1562	Max VG
		High VG
		100 % VG
		90/10 VG/PG
		80/20 VG/PG
Balanced but high VG	700	75/25 VG/PG
		70/30 VG/PG
		60/40 VG/PG
Balanced	393	50/50 VG/PG
Balanced but high PG	166	40/60 VG/PG
		30/70 VG/PG
		25/75 VG/PG
High PG	122	20/80 VG/PG
		High PG
		Max PG

Throat hit is the feeling of smoke hitting the back of the throat [21]. E-cigarette users, who are former or even current traditional cigarette users, are more likely to prefer this feeling for satisfaction and sensation fulfillment [21, 22]. However, users who pick up smoking behavior by using e-cigarettes may be more likely to find the throat hit is less desirable or comfortable. The throat hit was the most common symptom inferred from the data we collected. Thus, we first studied the throat hit among e-cigarette users for our symptoms analysis. We used the phrase “throat hit” to identify posts that are related to this symptom.

E-juice is basically made of 4 components: VG, PG, nicotine, and flavoring [4]. Each component could produce some direct effect or side effect on the user’s throat hit. Nicotine is the major source of throat hit. Some early traditional tobacco studies revealed that nicotine level could be a significant factor for producing a throat hit [23–25]. Thus, the higher concentration of nicotine e-juice contains, the stronger the throat hit is. In this research, based on the information reported by e-cigarette users, we classified nicotine level into 4 categories: 0 mg, 1–6 mg, 7–15 mg, 15 or more mg. PG is another source of throat hit. Although the FDA considers PG to be safe, some research and users’ reports have shown that PG could probably introduce more throat hit [26, 27]. The FDA has also deemed VG safe [28], although no research has shown VG as a source of throat hit. Flavors could also be responsible for throat hit. For example, some posts in our dataset mentioned that menthol flavor could produce a feasible throat hit for former traditional cigarette users. A few other substances could also cause throat hit. For instance, some strawberry flavors were reported to be alcohol based, which could make the taste of e-juice really harsh and be considered as a sort of throat hit. In order to capture the general characteristics among each flavor category, we filtered posts with information of VG/PG, nicotine, and throat hit and then calculated the average value of each variable. Therefore, the relationship between throat hit and e-juice components was studied.

Finally, based on the research in Hua et al. (2013) [29], which included a total of 10 systems and 405 different symptoms, we followed their disease categories and looked up the symptoms in our dataset,a total of 9 systems of 25 symptoms were identified and analyzed in detail based on e-juice components and user sentiments.

To accurately analyze the relationship between symptoms and components of e-liquid, we manually identified the positive and negative sentiment of symptoms. Namely, we firstly examined the overall user sentiments among all the symptoms. Each post was classified as positive or negative piece. Positive sentiment referred to happiness, praise, affection, enjoyment, etc., while negative sentiment included sadness, aversion, uncomfortableness, pain, etc.

## Results

### Flavors and VG/PG ratios

**Fig. 1:**
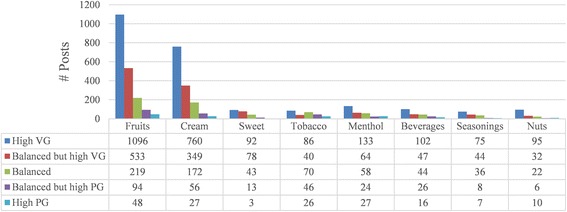
VGPG Ratio Distributions among Flavor Categories

**Fig. 2:**
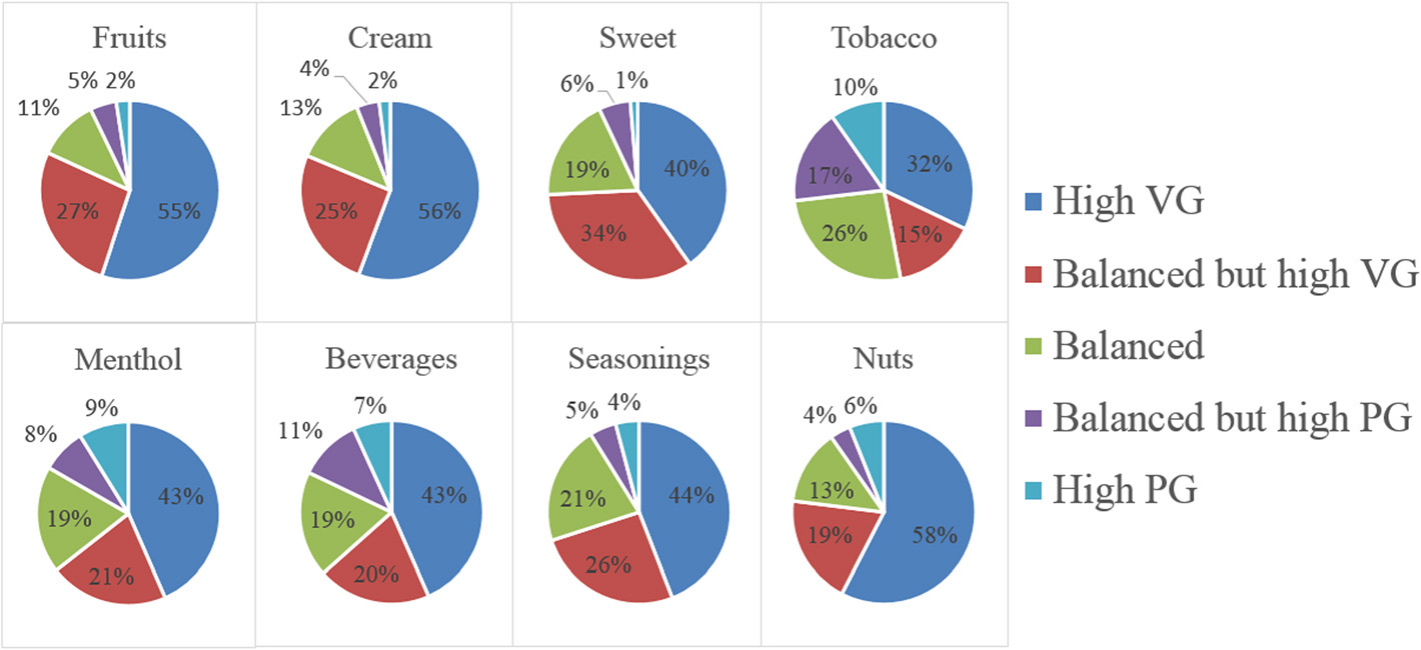
VGPG Ratio Percentage Comparisons

The distribution of all PG/VG ratios in different flavor categories is shown in Fig. 1. We have found that there are 3 typical patterns in the distribution, which are shown in Fig. 2. The fruits, cream, and nuts flavors could be considered similar flavors in terms of VG/PG ratio distributions. These flavors were sweet, smooth, and mild. E-cigarette users who preferred these kinds of flavors were more likely to enjoy the soft taste of e-cigarette smoking with thick vapor, which was exactly what VG liquid provides. More than 50 % of the posts related to fruits, cream and nuts were in high VG liquid. Only a tiny part of the posts were in high PG liquid. However, sweet and seasonings flavors were different. They had more posts with balanced VG/PG ratio. It is known that PG is thin in vapor but could deliver more flavor and throat hit [4, 6–8]. Users trying sweet and seasonings flavors were thus more interested in the pure flavor taste. Therefore, a higher ratio of PG liquid was preferred. The third type contained tobacco, menthol, and flavors. These three categories of flavors were more likely to be associated with high PG liquids. Tobacco flavor users tended to use e-cigarettes as a substitute for traditional cigarettes. Thus a fierce throat hit was an indispensable factor for those users. For this reason, the high PG posts showed a really high percentage (9.7 %) among tobacco flavor posts compared to other flavor categories (menthol scored second highest, 8.8 %; average was 3.5 %). As for beverage flavors, some e-cigarette users made their own tea or coffee e-juice. They used PG as the carrier to extract tea or coffee flavor into the liquid. Menthol flavors could provide a much fiercer throat hit than the other flavors, which we would like to show in the following discussion of throat hit and e-liquid components. Thus a higher PG concentration could help users to enjoy more throat hit.

It could be seen from Fig. 2 that the eight categories formed 3 groups. Fruits, cream, and nuts flavors were more VG based, while tobacco, menthol, and beverages were relatively more PG based. Sweet and seasonings flavors were in the middle.

### Throat hit and e-liquid components

**Fig. 3:**
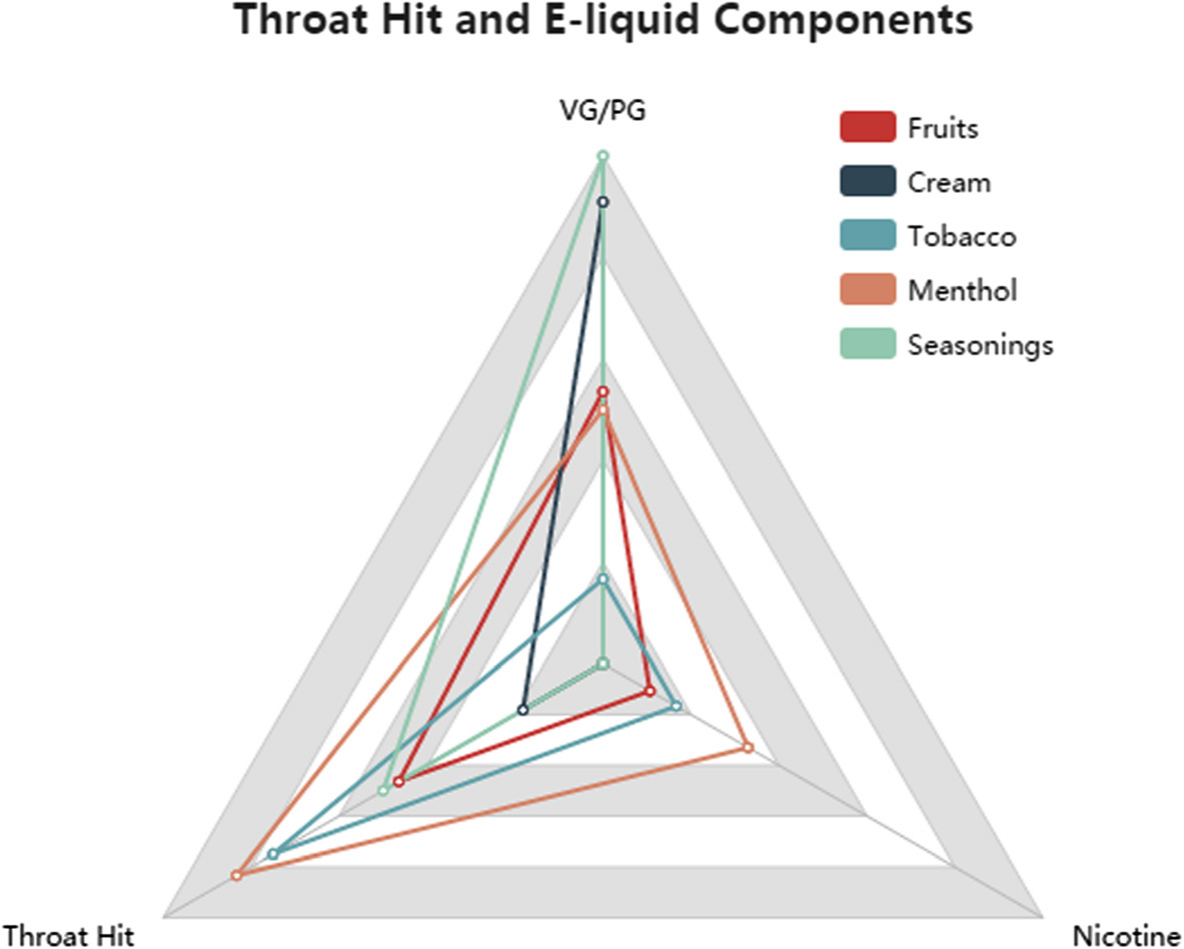
The Relationship between Throat Hit and E-liquid Components

To gain an understanding of the relationship among throat hit, VG/PG and nicotine levels of a variety of flavors, we counted the number of times that each flavor category, throat hit, VG/PG, and nicotine levels occur in the posts, the number of posts on throat hit, VG/PG, and Nicotine level for each flavor category (Fruits, Cream, Tobacco, Menthol, Seasonings) is 28, 11, 12, 6, 4, respectively. We drew a radar chart for illustration of the relationship between e-liquid components and throat hit, which is shown as Fig. 3. Typically, the fruits flavors were mild and not extreme in any of the variables. However, cream had a really high VG/PG ratio (90.91 %), which meant a very high VG solution was used in a typical cream flavor e-juice. Tobacco, which was the opposite of cream flavors, showed a high PG ratio (83.33 %), along with a high throat hit feeling (75 %). Menthol flavors also had a great throat hit feeling (83.33 %), but they were more varied in VG/PG ratios and nicotine levels. Seasonings flavors were mostly VG based (100 %), mild e-juices with 50 % throat hit.

From the patterns above, we could summarize that tobacco or menthol flavors, along with high PG ratio and high nicotine level, could probably produce very strong throat hits.

### Symptoms and e-juice components

The results are shown in Table 2, which is in the Appendix A. Example posts are excerpted for each of the symptoms in Table 3, in Appendix B as well.

The majority of users reported negative sentiments about symptoms, including cough and respiratory system problems/issues. Comments about sinus symptoms were generally positive. Neurological system symptoms were generally negative. While tingle and focus were positive, headache and fatigue were negative symptoms. Mouth and throat system symptoms tended to have positive sentiment. All other systems mostly reported negative sentiment. We added 2 more symptoms, allergy and cavity, both of which were negative.

Throat (*n* = 150) and mouth (*n* = 50) symptoms were the most common, probably because the most direct perception of feeling after vaping an e-cigarette was in the throat and mouth. In the previous section, our analysis on throat hit revealed the relationship between the degree of throat hit and VG/PG ratios, flavors, and nicotine levels. Many users enjoyed a slight throat hit to simulate the feeling of smoking traditional cigarettes. However, there still existed a large group of users who did not like throat hit.

Table 3 presented some examples of posts and replies that provided vivid descriptions of symptoms. We presented some further analysis for each symptom, based on the data collected. For instance, symptoms related to lungs were primarily related to high VG or balanced but high VG, of which fruits, cream, and menthol were the most common flavors. They usually had a nicotine level of 1–6 mg and 7–15 mg. Some users reported heaviness in lungs and shortness of breath due to some coffee extracts and oils. Some noticed lung tightness while exhaling, but others got a nice cooling sensation or a filled lung feeling, which were positive sentiments.

Most reported cough symptoms were related to balanced level to high VG level. Fruits, tobacco, menthol, and seasonings were the most common flavors, with nicotine levels of 1–6 mg and greater than 15 mg. People usually reported negative feelings such as harsh, straining, and burning along with a cough. This might be related to VG/PG ratio, flavors, or nicotine level. Interestingly, cinnamon was considered by some users as the reason for the cough, while watermelon flavor was thought to be soothing and reduce the cough reaction while vaping.

Sinus symptom was another interesting point of discussion. Most sinus symptom reports were positive. People reported that vaping e-cigarettes would be helpful to open up and/or clear sinuses.

Symptoms in neurological systems were mainly about headaches. High PG and fruits or seasonings flavors were most commonly reported. Nicotine levels were primarily over 15 mg, which was really high [30–35]. Some people reported horrible headaches, while some believed vaping helped headaches. Fatigue was an interesting symptom in this system. Users actually reported “vaper’s fatigue” or “vaper’s tongue”, which was a feeling when users could no longer get the feeling or taste of flavored e-juice. A high concentration of e-juice flavors might cause this dullness of sense of taste [36–38].

The most commonly reported symptoms were related to mouth and throat. All types of the VG/PG ratios, flavors, and nicotine levels were discussed by users. Symptoms related to throat could be further divided into 2 subgroups, throat hit and throat feeling, such as dry, harsh, sore, and burnt. Posts related to throat hit were more positive. Users were prone to discuss the combination of e-juice components that would create a comfortable throat hit. But the comfort level of throat hit was different for different people. While some enjoyed a slight throat hit, others preferred a moderate or strong throat hit. For instance, a user mentioned that menthol, strong tobacco, and other flavors would give more throat hit. Some people also commented that high PG is more likely to generate a strong throat hit. Another interesting finding was that some people reported negative feelings because they found the throat hit simply not strong enough. As for the dry, harsh, sore, and burnt throat feelings, most posts were negative. Some people expressed their concern about the relationship between PG and throat harshness, noting that drinking water or coffee might relieve the symptoms. Some flavors, such as citrus, sour, some cola flavors and some custards, were also considered as the reason for harshness. The positive posts provided some solutions to relieve the harshness, including trying smoother flavors or changing/different VG/PG ratios.

Mouth symptom posts frequently discussed fruits, cream, and menthol flavors, with nicotine levels of 1–6 mg. Most of these posts were negative, describing dryness and burning. For instance, a user used fruits blend flavor with 70/30 VG/PG ratio and felt dry mouth. This user said that marshmallow could be added to solve the problem. Another interesting finding on mouth symptoms was related to dripping. Dripping was simply taking a cartridge off the atomizer and putting 3–5 drops of e-juice directly onto the atomizer coil or bridge. Some people claimed dripping would produce a more direct and strong flavor sensation and throat hit. But some expressed concerns such as dripping would create “way too high” feeling in the mouth.

Digestive system symptoms were related to digestion, stomach, and heartburn. Data was sparse but the reports from users could still be meaningful since they were based on real-life experiences. One person reported “terrible and embarrassing” digestive problems, while another one said “this (LemonLock) juice literally made me sick to my stomach.” Heartburn, which is not as common as stomachache, was reported by users in conjunction with high VG and cucumber melon flavor.

Symptoms in sensory, chest, and immune categories were mostly negative. Some people reported feeling cold after vaping, and others had chest-tightness and burning feelings.

Allergy and cavity were also included in our analysis. We found that an allergy, or sensitivity, was common and important. PG sensitivity was one of the most important allergies, which would produce symptoms in the throat, heart, or stomach. A person with nut allergy commented that the smell of TFA peanut butter caused a nauseous feeling. Cavity was also of interest. A user complained about having 5 cavities after trying e-cigarettes. The sweet juice was considered the cause of the cavities.

## Discussion

To the best of our knowledge, this is the first systematic study of e-liquid components based on data collected from Reddit. In our previous research, we used Reddit as a social media data source for e-cigarette research. E-liquid flavor topics, including classification, popularity, and characteristics, were studied in [20]. In this study, 3 major components, including VG/PG ratio, flavors, and nicotine level, were analyzed, as well as their inter-connecting relationships. Disease symptoms were excerpted from the dataset and connected with these components. We believe the information from real-life user experiences is useful for further examination of e-cigarette use and for policy makers considering regulations.

For instance, these real user experiences would be of great importance for acquiring basic knowledge of vaping. Users are able to learn from one another so as to avoid negative experiences. The posts about PG sensitivity serve as a warning for e-cigarette users with allergies. On the other hand, positive posts could attract new users. For example, sinus patients might consider trying e-cigarettes after reading several posts declaring sinus clearing satisfaction. Sharing these symptoms and patterns of e-juice components plays an important role in the e-cigarette community.

For e-cigarette vendors, the user experiences reflect preferences, which is the core of promotion and development considerations. Popular patterns could be detected and predicted by the data from social media.

For medical research, real user experiences could be an important direction for further study. Several frequent or interesting symptoms identified from our study could be examined deeply based on medical and clinical methodologies. For regulatory departments, our study have found several links between negative experience and e-juice components, which provides useful references for policy makers.

The prevalence of e-cigarette use raises new concerns about this product, which is advertised as an approach to help smokers quit smoking, while the large variety of sweet flavors could increase appeal to adolescents and young adults. Our study reveals the components of e-juice and suggests some possible relationships between those components and vaping symptoms. We appeal for more clinical examination of e-juice components to avoid negative symptoms as much as possible.

### Contributions, limitations, and future research

#### Contributions

E-cigarette is an emerging product considered as a substitute for tobacco cigarette [39–41]. Although plenty of research has been done in this field, great gaps still need to be filled. Our research utilized data from Reddit to study the components of e-juice, and the symptoms related to these components. We believe the detailed examination of the 3 components, specifically, VG/PG ratios, flavors, and nicotine levels, in this study would be helpful to form a better understanding of this product.

This is the first research to comprehensively classify all of the VG/PG ratios, flavors, and nicotine levels. We believe the category framework used in our research could be helpful in other studies to generate meaningful insights.

This is also the first study to examine the throat hit in details. Menthol and tobacco flavors, along with high PG ratio and high nicotine level, are summarized to be more likely to generate strong throat hit. From user self-reported contents, we have revealed that some people enjoy strong throat hit, while some others prefer vaping experience with no throat hit. Despite the preferences, people have positive or negative sentiments towards this symptom.

Many other symptoms are also examined in this study. We take category of symptom systems from previous research to identify typical interactions between e-juice components and symptoms. Our findings suggest that, all of the VG/PG ratio, flavors, and nicotine level could be related to the symptoms of vaping, as well as the sentiment towards the symptoms. Some posts are further studied as examples.

#### Limitations

The dataset we have collected only covers the time window from January 1st 2010 to June 30th 2015. Posts and comments beyond this scope are not included in the dataset. Although including as much data as possible would provide a better and comprehensive understanding of e-cigarette, we believe the initial dataset with 493,994 records is large enough for obtaining 3605 e-liquid related posts in this study. We treat posts and comments as the same. That is to say, network structure of posts and comments are omitted, which could be valuable information for description and prediction model.

As for the data collection strategy, we miss the keywords “vape” and “vaping”, which are widely used among e-cigarette users. However, because of the huge amount of posts and comments, vape and vaping are also widely used in the posts we have collected. Thus, we still believe the validity of our research findings. In future research, we would like to expand the keywords set for a more comprehensive study.

In this study, we mainly provide descriptive results about our findings. In our future work, we will collect more data from cross social media and further examine the statistical correlation between e-liquid components and specific symptom based on prediction model such as regression model, classification model, etc.

Finally, Reddit does not provide demographic information for each user. Although we are really interested in including age and gender information into our study, we are not able to do that because they are not available.

#### Future research

Three streams of research can be further explored based on our findings. First of all, the decomposition of e-juice could be further analyzed. From our study, we notice that the components interact with each other to produce effect. For instance, the throat hit is related to tobacco or menthol flavors, high PG, and high nicotine level. The current clinical study of e-cigarette juice mostly isolates the effect of certain component. We propose that researchers in this area should be aware of the interactions among components. And future study should make use of these interactions to evaluate the effect of e-cigarette.

Second, this study has analyzed sentiments and symptoms with e-juice components. A more in-depth analysis of sentiments could reveal more information and patterns of e-juice composition, which should be studied in sophisticated algorithm. The symptoms, on the other hand, should be examined by clinical study to gain solid ground truth of the effect of using e-cigarette. Both of them are valuable research topics. Our study suggests that the e-juice components should be taken into consideration when these two type of research is conducted.

Finally, user demographic information should be considered to be included in the research of e-cigarette. Our finding only suggests some general relationships between symptoms and e-juice components. However, with the help of demographic information, we could figure out which sub-group of population are easily caught by certain symptoms. Then some specific regulation could be made to aim at these vulnerable people. While survey could be used to collect demographic information directly, some information techniques could be used to derive the age and gender indirectly based on the data from social media. The algorithm should utilize the knowledge of this area to attain a high precision rate.

## Conclusions

In conclusion, this is the first study utilizing Reddit to study the components of e-juice. Our finding suggests that Reddit is a good social media platform to study e-cigarette phenomena from the perspective of information researchers. We systematically categorized VG/PG ratios, flavors, and nicotine levels, which were 3 major components in e-juice. Symptoms, especially throat hit, were analyzed based on the findings of e-juice components. We found that menthol or nicotine flavors, along with high PG and high nicotine level, would be the probable reason for strong throat hit. Some other symptoms were studied in some cases. Our findings of e-juice component categories provides a possible framework for future research to adopt. Finally, information revealed in this study could be utilized by e-cigarette users to understand the patterns of e-juice and symptoms, by vendors to choose appropriate recipes of e-juice, and by policy makers to propose new regulations.

## Abbreviations

FDA, the U.S. Food and Drug Administration; PG, propylene glycol; VG, vegetable glycerine

## Appendix A

**Table 2:** Symptoms summary (n is the number of posts)

System	Symptom	VG/PG	Flavor	Nicotine	Sentiment
Respiratory	Lung (*n* = 25)	High VG (*n* = 11)	Fruits (*n* = 14)	0 mg (*n* = 1)	Positive (*n* = 12)
		Balanced but High VG (*n* = 6)	Cream (*n* = 4)	1-6 mg (*n* = 6)	Negative (*n* = 13)
		Balanced (*n* = 3)	Sweet (*n* = 2)	7-15 mg (*n* = 4)	
		Balanced but High PG (*n* = 2)	Tobacco (*n* = 2)	>15 mg (*n* = 1)	
		High PG (*n* = 1)	Menthol (*n* = 4)		
			Beverage (*n* = 2)		
			Seasonings (*n* = 2)		
			Nuts (*n* = 0)		
Respiratory	Cough (*n* = 16)	High VG (*n* = 3)	Fruits (*n* = 7)	0 mg (*n* = 1)	Positive (*n* = 4)
		Balanced but High VG (*n* = 4)	Cream (*n* = 1)	1-6 mg (*n* = 5)	Negative (*n* = 12)
		Balanced (*n* = 4)	Sweet (*n* = 1)	7-15 mg (*n* = 2)	
		Balanced but High PG (*n* = 1)	Tobacco (*n* = 3)	>15 mg (*n* = 4)	
		High PG (*n* = 1)	Menthol (*n* = 3)		
			Beverage (*n* = 0)		
			Seasonings (*n* = 2)		
			Nut s (*n* = 0)		
Respiratory	Phlegm (*n* = 1)	Balanced but High PG (*n* = 1)	Fruits (*n* = 1)		Negative (*n* = 1)
Respiratory	Sinus (*n* = 5)	Balanced but High VG (*n* = 1)	Cream (*n* = 1)	0 mg (*n* = 1)	Positive (*n* = 4)
		Balanced (*n* = 2)	Tobacco (*n* = 1)	7-15 mg (*n* = 1)	Negative (*n* = 1)
		High PG (*n* = 1)	Menthol (*n* = 3)		
Neurological	Headache (*n* = 9)	High VG (*n* = 1)	Fruits (*n* = 1)	0 mg (*n* = 1)	Positive (*n* = 2)
		Balanced but High VG (*n* = 1)	Seasonings (*n* = 1)	>15 mg (*n* = 3)	Negative (*n* = 7)
		High PG (*n* = 3)			
Neurological	Sleep disorder (*n* = 1)		Cream (*n* = 1)		Negative (*n* = 1)
			Seasonings (*n* = 1)		
Neurological	Tingle (*n* = 2)		Menthol (*n* = 2)		Positive (*n* = 2)
Neurological	Fatigue (*n* = 2)	Balanced but High PG (*n* = 1)	Fruits (*n* = 1)	7-15 mg (*n* = 1)	Negative (*n* = 2)
			Sweet (*n* = 1)		
Neurological	Focus (*n* = 1)				Positive (*n* = 1)
Mouth & Throat	Throat (*n* = 150)	High VG (*n* = 62)	Fruits (*n* = 82)	0 mg (*n* = 14)	Positive (*n* = 80)
		Balanced but High VG (*n* = 20)	Cream (*n* = 49)	1-6 mg (*n* = 61)	Negative (*n* = 70)
		Balanced (*n* = 21)	Sweet (*n* = 8)	7-15 mg (*n* = 13)	
		Balanced but High PG (*n* = 14)	Tobacco (*n* = 11)	>15 mg (*n* = 10)	
		High PG (*n* = 10)	Menthol (*n* = 24)		
			Beverage (*n* = 2)		
			Seasonings (*n* = 10)		
			Nuts (*n* = 1)		
Mouth & Throat	Mouth (*n* = 50)	High VG (*n* = 17)	Fruits (*n* = 34)	0 mg (*n* = 4)	Positive (*n* = 36)
		Balanced but High VG (*n* = 16)	Cream (*n* = 13)	1-6 mg (*n* = 21)	Negative (*n* = 12)
		Balanced (*n* = 6)	Sweet (*n* = 1)		
		Balanced but High PG (*n* = 4)	Tobacco (*n* = 2)		
		High PG (*n* = 0)	Menthol (*n* = 6)		
			Beverage (*n* = 2)		
			Seasonings (*n* = 2)	7-15 mg (*n* = 2)	
			Nuts (*n* = 1)	>15 mg (*n* = 1)	
Mouth & Throat	Tongue (*n* = 28)	High VG (*n* = 15)	Fruits (*n* = 18)	0 mg (*n* = 1)	Positive (*n* = 22)
		Balanced but High VG (*n* = 8)	Cream (*n* = 11)	1-6 mg (*n* = 11)	Negative (*n* = 6)
		Balanced (*n* = 2)	Sweet (*n* = 6)	7-15 mg (*n* = 0)	
		Balanced but High PG (*n* = 2)	Tobacco (*n* = 0)	>15 mg (*n* = 0)	
		High PG (*n* = 0)	Menthol (*n* = 1)		
			Beverage (*n* = 0)		
			Seasonings (*n* = 3)		
			Nuts (*n* = 0)		
Mouth & Throat	Teeth (*n* = 3)	High VG (*n* = 1)	Fruits (*n* = 3)	1-6 mg (*n* = 1)	Positive (*n* = 3)
		Balanced but High VG (*n* = 2)	Cream (*n* = 1)		
Mouth & Throat	Lips (*n* = 4)	High VG (*n* = 3)	Fruits (*n* = 2)	1-6 mg (*n* = 1)	Positive (*n* = 3)
		Balanced but High VG (*n* = 1)	Cream (*n* = 1)		Negative (*n* = 1)
		Balanced (*n* = 1)	Seasonings (*n* = 1)		
Digestive	Digestion (*n* = 1)	Balanced but High PG (*n* = 1)	Tobacco (*n* = 1)		Negative (*n* = 1)
Digestive	Stomach (*n* = 3)	High VG (*n* = 1)	Fruits (*n* = 2)	1-6 mg (*n* = 1)	Negative (*n* = 3)
		High PG (*n* = 1)	Cream (*n* = 1)		
			Menthol (*n* = 1)		
Digestive	Heartburn (*n* = 1)	Balanced but High VG (*n* = 1)	Fruits (*n* = 1)		Negative (*n* = 1)
Sensory	Hearing (*n* = 1)	High PG (*n* = 1)			Negative (*n* = 1)
Chest	Pain (*n* = 1)		Menthol (*n* = 1)		Negative (*n* = 1)
Chest	Tightness (*n* = 3)	High PG (*n* = 3)			Negative (*n* = 3)
Chest	Other (*n* = 1)	High VG (*n* = 1)	Cream (*n* = 1)		Positive (*n* = 1)
		High PG (*n* = 1)	Menthol (*n* = 1)		Negative (*n* = 1)
Immune	Cold (*n* = 1)	Balanced but High PG (*n* = 1)	Fruits (*n* = 1)		Negative (*n* = 1)
			Tobacco (*n* = 1)		
			Menthol (*n* = 1)		
Circulatory	Pressure (*n* = 1)				Positive (*n* = 1)
Other	Allergy (*n* = 9)	High VG (*n* = 1)	Fruits (*n* = 1)	7-15 mg (*n* = 1)	Negative (*n* = 9)
		Balanced but High VG (*n* = 1)	Sweet (*n* = 1)		
		High PG (*n* = 7)			
Other	Cavity (*n* = 1)		Fruits (*n* = 1)		Negative (*n* = 1)
			Cream (*n* = 1)		

## Appendix B

**Table 3:** Example posts for symptoms

Symptom	Post
Lung	1. Before I even finished my first fill-up, I noticed a tightness in my upper lungs while exhaling, and an urge to cough that far exceeds the typical dripper’s throat-clearing feeling. I tried to power through it and keep vaping, but I felt like I was kind of dying and it kept getting worse.
	2. While Mount Baker Vapor juices were not for me, they do serve a place among newbie/cash-strapped vapers who vape primarily through a tank…Every juice is 100 % VG…The menthol isn’t strong. Just enough to give you a nice cooling sensation in your lungs and on the exhale…Rating: 4/5
Cough	1. I am a complete Noobie Vaper…1 x USA Blend Tobacco E Juice Baker Vapor @ 50/50 pg/vg with 24 mg nic strength…Now I also realize I was coughing from it but nothing else, and I was straining my throat so bad I was coughing up blood from the vaper, Not from coughing excessively just a Cough once or twice and not hard as if I was smoking a cigarette.
	2. I am new to vaping and decided to start with a vaporfi pro xl. For the most part it works fine except for the first 2–5 hits. They always taste really harsh, burnt, makes a lot of popping noises, and makes me cough quite a bit.
Phlegm	1. It just makes it more enjoyable and satisfies my sweet tooth. 50/50 is my go-to but I have yet to try a full VG mixture, as I have a slight PG allergy I think, where if I vape a 70/30 PG/VG for too long, my throat gets really dry and I get phlegmy from vaping. That’s just me though, some people do prefer the PG throat hit.
Sinus	1. Menthol tobacco – a perennial favourite, featuring sinus clearing satisfaction with that classic tobacco base note, sadly to my utter disgust… it works.
	2. I vape vanilla custard all the time. Immediately after mixing it tastes good but has an alcoholish after taste and can be very unpleasant and lock up my nostrils. (most flavor concentrates contain some amount of alcohol) After steeping it for a week its beautiful and every hit opens up my sinuses like nothing else (70%vg)
Headache	1. I’ve ordered some 0 mg Strawberry from a local vendor. It’s 75/25 VG/PG. I just started vaping it, and all I taste is very very sweet (excruciatingly painful sweet) sugar. It gave me a headache after a few drags.
	2. I find higher PG makes me vape less (due to the ‘fuller vape’ feeling from the increased throat hit), which in turn means less nicotine and less dehydration. Helped with my headaches.
Sleep disorder	1. I had made an vg mix with 5 g herb, and cinnamon cookies aroma, it was good but not so potent. It made me sleepy when I vape it, but now i use that to soak my herb into ago atomizer, and it gives a nice “cookie” taste :D
Tingle	1. Straw-menth (Nippy Strawberry) by Bumblebee Eliquids…This menthol coats the tongue, makes it tingle, but does not travel much further than that. It compliments the strawberry perfectly without it overpowering the overall flavour of the juice. Totally ADV material!
	2. On opening the bottle, you get a lovely natural aroma of pineapple. There are hints of coconut in there. A slight tingle in the nostrils tells you that there is also a touch of menthol.
Fatigue	1. After the first week or so I noticed the vapor losing flavor rapidly. A few google searches later and I had a list of ‘fixes’ for vaper’s tongue/fatigue/etc. 2. Winter Nip: This is based on a 19th century boiled sweet I’ve never heard of - I can understand why it’s an historic relic. Winter Nip is an incredibly strong menthol e-liquid, with an ice cooling effect on top of that… I guess if you have a bad cold or flu - or at a push, severe vapers fatigue - then Winter Nip might be up your street.
Throat	1. Sweet Complexity: PG/VG: MAX VG. Nic strength: 3 mg. Awful, but not amazing either. Rating: 6/10 - Sore throats beware this juice.
	2. Beeblebrox - 6 mg, 50/50 pg/vg. Rich pipe tobacco with a strong vanilla and savory spice kick. The tobacco flavor here is unique and quite unlike the generic “555” style Tobacco Absolute flavors commonly seen…Beeblebrox performs well with dense vapor production even at 50/50 pg/vg and has a mild yet satisfying throat hit. The room note is as pleasant as the juice itself…88/100
Mouth	1. Uuuuuuuuugh. I can taste what this was supposed to be, but it falls short. The fruit punch is there, but Bloodbath has an almost chalky mouth feel, and a weird chemical taste on the exhale…Rating: 3/5
	2. I was a dipper myself…I tried it! to my surprise 3 mg was actually enough for me though i havent dipped in a while. (I wouldnt mind upping to 4 or 5 mg). however first i tried just regular mix. the %’s you use for vaping…WAY TO HIGH for what you want in your mouth. tasted like perfume…80vg/20 pg…3 mg nic…actually wasn’t bad…of course I didnt swallow. just spit like its dip haha.
Tongue	1. All juice was steeped for two weeks, max VG, 6 mg…Sweet Complexity. Vendor Brown sugar and rum steal the finish at the end of the exhale, just lingering on your tongue long enough to let you know it’s there, but it doesn’t overpower the other flavors…10.5/10
	2. HELIX - 3 mg, 10/90 vg/pg…Described as melons, melons, and more melons. This was a good one…When I first juiced this one up, it was suuuuper smooth and delicious, but over time it sort of waffled between smooth and rough. Not rough like throat hit or coughing, but a kind of zingy mouthfee,l like when you eat too many lemons raw or how your tongue feels the day after you burn it on your tea. I’m going to chalk this roughness up to me wicking poorly though. Overall a good vape. 7.5/10
Teeth	1. Next up is a true southern taste known as Raspas…this flavor features crisp, ripe strawberries with a splash of tangy watermelon and a mild coconut finish…but now you can enjoy the flavor without your teeth chattering! Both of these flavors are from our Artisan Series of e-liquid with a 30 % PG and 70 % VG base.
	2. It’s a 30 PG/70 VG mix with a vanilla custard base with an aftertaste of some delicious blueberry. Often times I find the super sweet juices like this one have a “heavy” feel to them and make me feel like my teeth are going to rot out of my head, but not this one. It’s light and just the right amount of subtle.
Lips	1. Every juice is 100 % VG, and 3 mg nicotine…A primarily melon flavor with hints of mixed fruits and berries. From the wretched bowels of Scumdogs to your lips. A true Gwar classic. My Thoughts: Tastes like a green apple Jolly Rancher mixed with something that goes from pleasant to unpleasant with every other hit… Rating: 3/5
	2. One of the downsides from this is the heat buildup, it tends to get very very warm after chainvaping for a couple of minutes. If you build subohm coils like I did (0.4ohm dual) you’ll notice that chainvaping isn’t an option without burning your lips unless you use a delrin driptip.
Digestion	1. 100 % correct. I can’t vape much VG - I have terrible, terrible and embarrassing digestive problems when I go any higher than 25 or 30 % VG. I love the taste of Blu’s tobacco carts but they make me sick :(
Stomach	1. You must use around 90-95 % VG…TFA Menthol…I also liked some fruity flavors…I found 6 mg nic to be my speed, however!..oh, and in regards to gutting the e-juice. I never gut my dip, I preferred spitting and that translated into this method. but im sure it won’t hurt you, this stuff is food grade…however if you didn’t use to dip. i would steer clear from that method, as your stomach won’t be use to the nic.
	2. I tried “LemonLock” was not good at all! I loaded up my Delta 2 with it and in about 5 min of raping this juice literally made me sick to my stomach.
Heartburn	1. All high VG…Cucumber Melon. When I first tried this months ago in a Subtank, it was fantastic. It was my favorite juice and I went through a few 120’s. When I had it then, it was cool and the melon was mellow. The cucumber was a nice exhale, and it was a nice ADV. I recently tried dripping it after not having it for a few months and the flavor was there, but it was harsh. Not really a throat hit, but it felt like heartburn.
Hearing	1. I am dealing with similure inner ear symptoms and suspected it may be related to PG. I did talked to my ENT Doc last week at a follow up appointment after my CT scan reveled my sinuses are 90 % backed up with a pretty bad sinus infection. I went in about 3 weeks ago for hearing loss thinking it was just earwax build up.
Chest	1. All juices are Max VG and available in 0 mg, 3 mg, 6 mg, and 12 mg…The menthol is very well done and mild. Perfectly balanced with the flavors so that it compliments them without being overbearing. You get a nice cool sensation in your throat but no freezing of your face or chest.
	2. Hello vaping world! 1–2 pack a day smoker. And well, the history with ecigs… I was then told that a different VV mod may be a better choice to get more vapor, so I spent the money for TW’s e-lectron (same as the ecom) battery, but then I started to notice a tightness in my chest, gave it up again (thinking it was vapor itself).
Cold	1. I bought my first e cigarette 2 days ago and since yesterday, I have something that feels a bit like a cold but not really. My nose is kind of runny but not with thick stuff. It’s rather thin…It got better today… but it’s still annoying and I blast through paper towels like it’s chocolate. It doesn’t happen during the vaping. I have 55/35 PG/VG liquid. So, what’s going on here? Is that normal?
Pressure	1. Many people have found vaporizers to be a powerful smoking cessation tool. Those who have switched have lowered their blood pressure, improved respiratory health, and improved their lifespan.
Allergy	1. I’ve got a pretty severe PG sensitivity, makes my throat swell up after a while. I used a relatively cheap but tasty brand (George Dickel), I’ve got mint and lemongrass steeping but I think those will take more like a week.
	2. Selling the juice because I found out I really cant handle the PG (allergy). Most have a few ml missing due to trying them, a few are still full.
Cavity	1. Weird question here, is anyone else noticing their dental health has changed since using sweet liquids? Here’s some background: I’m an avid brusher, flosser and mouthwash user. I’ve never had a cavity before age 30 and suddenly my visit to the dentist last week revealed that I have 5 cavities!!! I always go to the dentist … I’ve not changed my diet habits or sweets intake and I take care of my teeth. In the past year, the only difference is that I started vaping (I know I’m weird, but I’ve never smoked actual cigarettes). So I’m thinking that maybe the sweet juice is causing cavities. I asked my dentist, but he doesn’t know a lot about vaping, but he thought maybe the sweet liquid could be a factor. I use a VP/PG mix from my local vape shop. It’s usually a banana/vanilla flavor. I know this is obscure, but I wanted to see if anyone else had this problem. I can’t find a lot online about it.
